# Utility of a New Artificial Dermis as a Successful Tool in Face and Scalp Reconstruction for Skin Cancer: Analysis of the Efficacy, Safety, and Aesthetic Outcomes

**DOI:** 10.1155/2020/4874035

**Published:** 2020-07-20

**Authors:** Fedele Lembo, Liberato Roberto Cecchino, Domenico Parisi, Aurelio Portincasa

**Affiliations:** Plastic and Reconstructive Surgery Department, Ospedali Riuniti-OORR, University of Foggia, Foggia 71100, Italy

## Abstract

Radical ablative surgery is the gold standard treatment of head skin cancer. The authors expose their experience with a new artificial dermis (Pelnac®), analyzing retrospectively the overall morbidity and aesthetic outcomes. 16 consecutive patients underwent two surgical procedures under local anesthesia. The first involved the tumor removal and application of the ADM. In the second, the exposed tissue was covered with a split-thickness skin graft. On follow-up (6 months), tumor recurrences, quality of scars (using the Vancouver Scar Scale), and patient reported outcomes (using FACE-Q Skin Cancer Module) were evaluated. 10 were males and 6 females, with a mean age of 73 years (61–89). The follow-up ranged from 12 to 48 months (mean: 30). The sites of skin tumor were scalp (12 cases), forehead (2), cheek (1), and zygomatic area (1). Nine patients underwent previous local surgery; two received radiotherapy. The average length of hospital stay was 3.2 days. The mean surface area of the defect was 59.15 cm^2^ (16.9–89.5). In three cases, the surgical bed was bone without periosteum. The malignant tumors excised were basal cell carcinoma (68.75%), squamous cell carcinoma (18.75%), malignant melanoma (6.25%), and sarcoma (6.25%). The mean operating time was 41 minutes for the first operation (25–55) and 34 for the second (25–48). No significant problems were observed and 15 patients (93.75%) had 100 percent intake of graft. The mean time of healing was 39 days (32–45). At 6 months post-op, no tumor recurrence. Satisfactory cosmetic and functional results were obtained in all patients as shown by the VSS Scale and FACEQ skin cancer module mean scores. We believe that the artificial dermis is a reliable alternative to flaps and should be considered an excellent option in head reconstruction for skin cancer, especially in critical patients (old, with large and deep defects, with recurrent tumors, required radiotherapy).

## 1. Introduction

Facial and scalp reconstruction following wide tumor resection is a continuous challenge for Dermatologist and Plastic Surgeons. In fact, the extent of resection (sometimes the galea and the periosteum removal is needed), the risk of tumor recurrence, and the probable adjuvant or neoadjuvant radiation therapy are the fundamental clinical problems to be solved.

An optimal reconstruction should guarantee a successful coverage and protection of exposed planes, maintaining a soft-tissue bulk and contour, with good aesthetical outcomes.

Moreover, the surgical coverage must be related to different factors such as patient's age, general status, clinical prognosis, type and side of the defect, the tissue surrounding the defect, adjuvant treatment, ability to care, and also cosmesis.

Skin grafts and local or free flaps (muscular, myocutaneous, myofascial, or fasciocutaneous) can be used for head reconstruction after oncologic demolition, but all present some disadvantages (as donor-site morbidity, significant long-lasting operative time and hospital stay, unstable results), especially in old patients with local advanced cancers, systemic diseases, or a prior history of recurring skin tumors.

So, when the autologous tissue cannot be used, the artificial dermis may provide a valid and reliable alternative with a low complication rate.

For more than 25 years, the artificial dermis has been successfully used in reconstructive surgery; up to now, however, there are still few reports of its use for reconstruction of complex facial and scalp defects, especially in older patients [1–7]. In particular, in the recent literature, there is no report of the use of Pelnac® (Gunze Corp., Osaka, Japan) in head reconstruction following skin cancer resection.

The authors present their clinical series of 16 consecutive patients who underwent wide resection of skin cancer of the head and successful reconstruction using a new artificial dermis (Pelnac®) at the Plastic Surgery Department of the University of Foggia-Ospedali Riuniti, Foggia, Italy.

The goal of this work is to explore the utility of this artificial dermis as a successful tool for postoncological reconstruction of wide and deep facial and scalp wounds, especially in older patients, that could not tolerate complex reconstructive procedures in general anesthesia, or need adjuvant radiotherapy, analyzing the overall morbidity of the surgical technique and aesthetic outcomes.

## 2. Patients and Methods

In this study, the authors collected the data of 16 consecutive patients who underwent wide excision of malignant skin tumor of the head and reconstruction using the artificial dermal matrix (ADM) marked Pelnac®, at the Plastic Surgery Department in Ospedali Riuniti OORR, Foggia, Italy, between January 2018 and December 2019.

They were reviewed regarding their age, clinical history, tumor site, history of previous surgery, chemotherapy, or radiation therapy (see Table 1).

All patients provided written consent and completed a preoperative FACE-Q Skin Cancer Module. The study was approved by the Local Medical Ethics Committee in accordance with the Helsinki Declaration of 1975, as amended in 2008.

The patients underwent two surgical procedures, performed under local anesthesia (using lidocaine or mepivacaine with adrenalin) and, if necessary, sedation. Perioperative antimicrobial prophylaxis with 2 grams of cefazolin was used in every patient.

The first stage involved the tumor removal, according to the guidelines, and the application of the dermal regeneration template. If the skin cancer infiltrated the calvaria, osteotomy was performed in order to reach the bleeding points. After oncological resection, the device (with the same size of defect area) was applied and secured in place using a tie-over with Vaseline gauzes.

**Figure 1:**
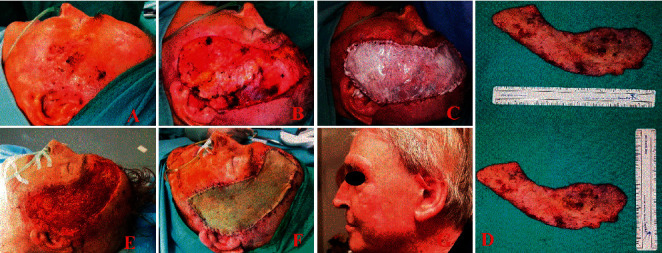
A clinical case of a 73 yrs old man, with multiple recidivate BCC of face, underwent wide excision and reconstruction with Pelnac®. (a) Preoperative view; (b) wide and deep excision of BBCs; (c) Pelnac® on side; (d) surgical specimens; (e) wound bed after silicon layer peeled off; (f) split-thickness skin graft; (g) follow-up at 6 months.

The first dressing change with tie-over removal was performed on postoperative days five to seven. The wound bed was inspected, thereafter, every week to detect the well-vascularized neodermis under the silicone layer, and wet-to-dry dressings were performed. In the second procedure (generally three weeks after), the removal of the silicone layer was conducted and the exposed tissue was covered with a split-thickness skin graft (from 0.4 to 0.6 mm) took from thigh or forearm. It was also fixed and tied over (see Figure 1).

At post-op follow-up (7-15-30 days), the area was examined for wound healing and wet-to-dry dressings were performed.

The type of tumor, the size of the surgical defect, the first and the second operation time, the length of hospitalization, the healing time, and the complications were recorded (see Table 2).

On subsequent follow-up (6 months), the authors evaluated tumor recurrences, quality of scars (using the Vancouver Scar Scale, VSS), and patient reported outcome (using FACE-Q Skin Cancer Module).

The Vancouver Scar Scale analyzes 5 variables: vascularity, thickness, pliability, pigmentation, and relief. Those parameters are expressed with a numerical value, giving a range from 0 (normal skin) to 13 (worst scar imaginable).

The FACE-Q Skin Cancer Module is a validated, patient reported outcome instrument used to quantify health-related quality of life after facial skin cancer surgery [8–11]. Data from FACE-Q Skin Cancer Module were converted into an equivalent Rasch transformed score using the conversion table, giving a range from 0 (the worst outcome) to 100 (the best outcome).

The authors present summary statistics as means with standard deviation (Std) and medians with a range for continuous variables and as frequencies and percentages for categorical variables. The continuous data (age and defect size) were assessed for normality of distribution using a Kolmogorov–Smirnov test that revealed a normal Gaussian distribution. A comparison of the means was performed using the *T*-Student test. An expert biostatistician performed the statistical analysis using Statistical Package for Social Sciences (SPSS version 16.0). A value of *p* less than 0.05 was considered statistically significant.

## 3. Results

A total of 16 patients underwent reconstruction of tumor resection defects with Pelnac® dermal template at the Plastic and Reconstructive Surgery Department, University of Foggia, Ospedali Riuniti, Foggia, Italy, from January 2018 to December 2019.

10 (62.5 percent) were male and 6 (37.5 percent) were female. The mean age of patients was 73 years (median: 72.5; range: 61–89; Std: 7.28). The follow-up ranged from 12 to 48 months (mean: 30; median: 29; Std: 11.06).

The site of wide malignant skin tumor of the head was various: scalp (12 cases), forehead (2), cheek (1), and zygomatic area (1).

Neoadjuvant radiation therapy was administered to one patient (6.25 percent) with a wide tumor of the scalp, whereas only one patient (6.25 percent) received postoperative RT.

Nine patients (56.25 percent) underwent to previous surgical procedures: 4 patients had a prior diagnostic biopsy, and 5 had the previous excision of local skin cancer.

Mean surface area of defect after tumor excision was 59.15 cm^2^ (median: 61.6; range: 16.9–89.5 cm^2^; Std: 20.88).

In three cases (18.75 percent), the surgical bed was bone without periosteum.

The malignant tumors excised were 11 basal cell carcinoma (68.75%), 3 squamous cell carcinoma (18.75%), 1 malignant melanoma (6.25%), and 1 sarcoma (6.25%).

The mean operating time was 41 minutes for the first operation (median: 41.5; range: 25–55; Std: 7.47) and 34 minutes for the second operation (median: 35; range: 25–48; Std: 6.8).

The average length of hospital stay was 3.2 days, ranging from two to five days (median: 3; Std: 0.8).

After the first operation, no significant problems were observed.

There were no complications associated with the Pelnac® dermal regeneration template application. Only two patients (12.5%) experienced minimal silicone detachment before skin grafting. In these cases, the unattached silicone layer was excised, and advanced daily dressing changes were carried out.

A split-thickness skin graft was applied in all the patients roughly from 3 to 4 weeks after Pelnac® application (mean 24 days; range: 21–32), when clinically the defect area revealed a well-vascularized neodermis.

15 patients (93.75%) had 100 percent take of their graft, but one case (6.25%) had approximately 95 percent take (there was a partial skin graft necrosis with spontaneous healing without complications). The patient who received adjuvant radiation therapy developed mild radiation-related complications, including blister formation and minimal graft ulceration. On subsequent follow-up visits, that patient healed well, without residual deficits. Spontaneous healings without complications were observed in donor-site areas, with good satisfaction of the older patients.

The mean time of healing was 39 days (median: 39; range: 32–45; Std: 3.54).

At 6 months post-op, no tumor recurrence was observed and scar appearance was assessed by an expert blinded Plastic Surgeon using the VSS Scale. The mean score assessed was 4 (range: 3–8, Std: 1.26); it shows that significant satisfactory cosmetic results (graft flat, pliable, with normal pigmentation and vascularization, and not fixed to the underlying bone) were obtained in all patients.

The results from the FACE-Q Skin Cancer Module are shown in Table 3.

Overall, both before and after surgery, the patients were satisfied with their facial appearance (from a mean preoperative score of 76 to mean postoperative score of 74 at 6 months), were not disturbed by scars (from a mean preoperative score of 74 to mean postoperative scores of 73 at 6 months), and had low levels of appearance related psychosocial distress (from a mean preoperative score of 27 to mean postoperative scores of 25 at 6 months). They were satisfied with the information they received. The mean score regarding worry about cancer decreased after excision (from a mean preoperative score of 55 to mean postoperative score of 37 at 6 months). Moreover, the adverse events related to facial skin cancer, including bleeding, itching, and pain, decreased after excision (from a mean preoperative score of 15 to a mean postoperative score of 13 at 6 months). The patients improved sun protection behaviors, such as the use of sunscreen, hats, and protective clothing, after diagnosis of cancer (from a mean preoperative score of 12 to a mean postoperative score of 17 at 6 months).

## 4. Discussion

Head reconstruction after skin cancers resection represents a continuous challenge for Dermatologist and Plastic Surgeons.

Excluding the spontaneous healing and the direct suture, the use of full-thickness skin grafts is a good option only for smaller defects. Larger wounds need split-thickness skin grafts (STSG), but they may associate with recurrent ulcerations, especially when the radiotherapy is necessary, and they need a good vascularization of the wound bed.

Unlike STSG, the local and regional flaps can provide more stable coverage with similar skin, but they cannot be considered for wide wounds or for the areas compromised by previous reconstructions and/or irradiation.

The free tissue transfer (as large fasciocutaneous flaps) offers a durable, pliable, reliable, and well-vascularized tissue and, thus, remains the mainstay repair option for large full-thickness scalp or facial defects, especially when postoperative radiation therapy is necessary.

However, the free tissue transfers are associated with significant donor-site morbidity and require expertise, a dedicated nursing care and a prolonged operative time and hospitalization.

Furthermore, the patient's age has been identified as a significant predictor of postoperative morbidity; therefore, these prolonged and complicated procedures in elderly patients may not be advisable.

The authors present their approach to face and scalp reconstruction after tumor resection, using a new tissue-engineered dermal regeneration template (marked as Pelnac®). The device was used to form neodermis, before STSG is applied 3 weeks later, improving the take and natural look of the reconstructed site. This artificial dermis, first described by Suzuki et al. [12, 13], is an acellular double-layer synthetic construct consisting of an outer silicone (polysiloxane) semipermeable membrane and an inner 3D porous matrix of collagen.

The outer layer serves as an epidermal substitute and provides mechanical and infective protection, modulates heat and moisture, and prevents hypergranulation. The inner layer, composed of porcine tendon type I collagen purified (atelocollagen), is an acellular dermal scaffold that promotes cellular ingrowth [14]. The dermal matrix of collagen is almost identical to endogenous collagen which results in poor antigenic response and a lower rate of rejection. In fact, it is gradually replaced by host (endogenous) collagen by natural wound healing mechanism (local flogosis, cell infiltration, and neoangiogenesis), forming a new dermal layer. This neodermis is consistent, elastic, and resistant to collagenases [15–20].

At approximately three weeks, when well-vascularized neodermis is usually formed, the silicone layer may be peeled off and replaced with an autologous thin split-thickness skin graft.

From the first studies of Suzuki on the possible applications of the new substitute [21], several authors produced about a dozen publications on its use, generally posttrauma [22–28], or for the correction of burn scars [29–31], or postremoval of giant nevi [32, 33], or for ulcers repair [34, 35].

To date, there are no publications of its use in head reconstruction after skin tumor resection.

The use of artificial dermis offers many advantages, such as the immediate availability, the possibility to cover large defects, the minimal donor-site morbidity, the good cosmetic results with optimal contouring, and the early recovery. On the other hand, the major disadvantage of the artificial dermis use is the need for a second operative procedure; however, both the procedures are fast and can be carried on in local anesthesia.

In our clinical series, the successful application of the artificial dermis (Pelnac®) for coverage of complex postoncological large defects of the head, especially in “complicated” older patients, was demonstrated with satisfactory cosmetic results and good patient outcomes.

The main reason for using artificial dermis in these patients was to provide a durable coverage, thicker than direct skin grafting, in older patients that could not tolerate complex and long reconstructive procedures as free flap transfers. Moreover, this technique allows early detection of local tumor recurrence and, therefore, its early removal. Furthermore, as opposed to local flap coverage, this procedure preserves the orientation of original surgical margins. So, this technique may be used as the initial phase of definitive treatment to provide a temporary wound closure (with instantaneous skull protection preventing infection and moisture loss) until the final pathologic examination results are known. Concurrently, the use of artificial dermis does not preclude further use of tissue transfer if necessary.

The quality of the surgical bed also plays an important role in the satisfactory vascularization of the artificial dermis. Nowadays, there are few experimental and clinical data on its application on bare calvaria.

In four cases (25 percent) of our patients, Pelnac® was applied, with satisfactory results, directly on bone without periosteum as it was infiltrated by tumor. In fact, even if the wound bed is avascular, the ADM guaranteed the peripheral neoangiogenesis into the dermal matrix in a centripetal way.

Moreover, adjuvant or neoadjuvant radiotherapy is a possible treatment in head skin cancers. Based on our limited experience, although the results are good and encouraging, caution should be exercised when applying Pelnac® in areas of previous or potential irradiation.

In our experience, in a patient (6.25 percent), the surgical site (scalp) had been irradiated prior to tumor resection; and in a patient (6.25 percent), however, the entire bed was irradiated after surgery. The only complication observed was a superficial skin graft necrosis, which healed spontaneously. No patient required surgical revision. The dermal substitute held up extremely well and skin grafts applied to the neodermis did not contract and often demonstrated good pliability on follow-up. Moreover, all patients, using the FACEQ skin cancer module, revealed satisfaction with their outcomes and the care received.

## 5. Conclusions

The reconstruction of scalp and face after wide skin cancer resection remains a formidable clinical challenge. To our knowledge, this is the first report in literature of the use of Pelnac® in head skin cancer surgery and of the use of the FACEQ skin cancer module to analyze the patient outcomes.

The current techniques of reconstruction are skin grafting and local or free tissue flaps. Although tissue transfer is a reliable method of reconstruction, it is often associated with severe donor-site morbidity, many aesthetic complications, and a high rate of failure, especially in older patients with severe pathologies, recurrent skin cancers, and the necessity of adjuvant or neoadjuvant radiotherapy. In our experience, Pelnac® is particularly suitable to repair large scalp defects, e.g., in bald men suffering extensive skin cancer. In fact, on the basis of our clinical series, it appears that the use of this artificial dermis, associated with S.T.S.G., results in good cosmetic outcomes with minimal donor-site morbidity and complications, and good patient satisfaction.

In particular, in our study, using the FACEQ skin cancer module, we found that the patients were satisfied with their outcome and the care received, with the reduction of cancer worry and psychosocial distress.

Although these data are just preliminary, we believe that the artificial dermis is a reliable alternative to flaps and should be considered an excellent option in head reconstruction for skin cancer, especially in older critical patients with large and deep defects, in patients with recurrent tumors, and in patients who require radiotherapy. Further future research is needed to increase the number of cases treated and to compare the results with those obtained with other ADMs.

## Figures and Tables

**Table 1:** Preoperative clinical data of the patients who underwent surgery.

ID	Tumor type	Defect size (cm^2^)	First operation time (min.)	Second operation time (min.)	Length hospitalization (days)	Healing time (days)	Complications	Follow-up (months)
1	BC	83.7	50	35	1 + 3	45	No	12
2	BC	79.7	45	38	1 + 2	40	No	48
3	SC	65.2	40	32	1 + 2	39	No	40
4	BC	24.7	28	25	1 + 1	37	No	33
5	BC	58.9	40	35	1 + 2	38	No	18
6	BC	45.2	37	38	1 + 2	34	No	23
7	BC	63.7	40	40	1 + 3	37	No	27
8	BC	72.1	44	45	1 + 3	43	No	31
9	MM	16.9	25	25	1 + 1	32	No	43
10	BC	56.2	43	30	1 + 2	39	No	45
11	BC	67.4	45	35	1 + 2	40	No	39
12	SC	32.2	38	30	1 + 1	36	No	19
13	BC	89.5	55	48	1 + 4	45	Partial skin graft necrosis	27
14	BC	78.3	48	37	1 + 3	40	No	37
15	SRC	59.5	40	28	1 + 2	41	No	20
16	SC	53.3	45	26	1 + 2	38	No	19

DM: Diabetes Mellitus, MDT: Multidrug Therapy, S: Smoke.

**Table 2:** Postoperative data collected.

ID	Sex	Clinical history	Age	Tumor site	Neoadjuvant therapy	Adjuvant therapy	Previous local surgery
1	F		72	SCALP	No	No	Yes
2	M	DM, MDT, S	89	SCALP	No	No	No
3	M	MDT	66	SCALP	No	No	Yes
4	M	DM	67	CHEEK	No	No	Yes
5	F	S	82	SCALP	No	No	No
6	M	MDT	79	SCALP	No	No	Yes
7	M	S	77	SCALP	No	No	Yes
8	F	DM	75	SCALP	No	No	No
9	M		68	FOREHEAD	No	No	No
10	M	MDT	61	SCALP	No	No	No
11	F		65	SCALP	No	No	No
12	M		75	SCALP	No	No	Yes
13	F	S	73	SCALP	RT	No	Yes
14	M	MDT	81	FOREHEAD	No	No	Yes
15	F	DM,MDT	72	SCALP	No	RT	Yes
16	M		69	ZYGOMATIC	No	No	No

BC: Basal cell carcinoma, SC: Squamous cell carcinoma, MM: Malignant melanoma, SRC: Sarcoma.

**Table 3:** Mean FaceQ skin cancer module scores.

FACEQ category	Scale	Preoperative score	Postoperative score (6^th^ mo.)	*p* value
Satisfaction with facial appearance	0–100	76	74	0.03
Satisfaction with information	0–100	100	98	0.001
Cancer worry	0–100	55	37	0.012
Psychosocial distress	0–100	27	25	0.03
Appraisal of scars	0–100	74	73	0.047
Adverse events	10–40	15	13	0.001
Sun protection behavior	5–20	12	17	0.001

## Data Availability

The data used to support the findings of this study are available from the corresponding author upon request.

## Supplementary Materials

Supplementary Materials Pre-, intra-, and postoperative photos and a videoclip of Pelnac® application after resection of wide BCC in zygomatic area.
