# Supplementary figures and images for: Utility of a New Artificial Dermis as a Successful Tool in Face and Scalp Reconstruction for Skin Cancer: Analysis of the Efficacy, Safety, and Aesthetic Outcomes
Source: Dermatol Res Pract. 2020 Jul 20;2020:4874035. doi: 10.1155/2020/4874035 (PMC7388001; doi:10.1155/2020/4874035)

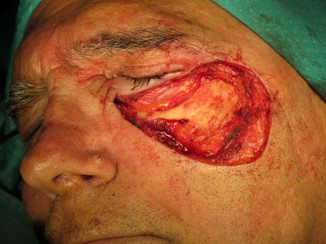

Supplement: Supplementary Materials — Pre-, intra-, and postoperative photos and a videoclip of Pelnac® application after resection of wide BCC in zygomatic area. [file 4874035.f1.zip › 4874035.f1/intra.jpg]

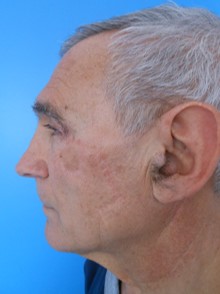

Supplement: Supplementary Materials — Pre-, intra-, and postoperative photos and a videoclip of Pelnac® application after resection of wide BCC in zygomatic area. [file 4874035.f1.zip › 4874035.f1/post.jpg]

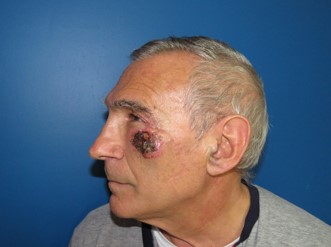

Supplement: Supplementary Materials — Pre-, intra-, and postoperative photos and a videoclip of Pelnac® application after resection of wide BCC in zygomatic area. [file 4874035.f1.zip › 4874035.f1/pre.jpg]
